# Insights into Adaption and Growth Evolution: Genome–Wide Copy Number Variation Analysis in Chinese Hainan Yellow Cattle Using Whole–Genome Re–Sequencing Data

**DOI:** 10.3390/ijms252211919

**Published:** 2024-11-06

**Authors:** Ziqi Zhong, Ziyi Wang, Xinfeng Xie, Deyou Pan, Zhiqing Su, Jinwei Fan, Qian Xiao, Ruiping Sun

**Affiliations:** 1 Institute of Animal Husbandry and Veterinary Research, Hainan Academy of Agricultural Sciences, Key Laboratory of Tropical Animal Breeding and Epidemic Disease Research, Haikou 571100, China; zhongziqi2021@163.com; 2 School of Tropical Agriculture and Forestry, Hainan University, Haikou 570228, China; wang1450887911@163.com (Z.W.); xinfeng2021@126.com (X.X.); pandeyou26@163.com (D.P.); 24210905000006@hainanu.edu.cn (Z.S.); 24220951330001@hainanu.edu.cn (J.F.)

**Keywords:** Hainan yellow cattle, CNV, population genetic structure, selection signatures, whole–genome sequencing

## Abstract

Copy number variation (CNV) serves as a crucial source of genomic variation and significantly aids in the mining of genomic information in cattle. This study aims to analyze re–sequencing data from Chinese Hainan yellow cattle, to uncover breed CNV information, and to elucidate the resources of population genetic variation. We conducted whole–genome sequencing on 30 Chinese Hainan yellow cattle, thus generating 814.50 Gb of raw data. CNVs were called using CNVnator software, and subsequent filtering with Plink and HandyCNV yielded 197,434 high–quality CNVs and 5852 CNV regions (CNVRs). Notably, the proportion of deleted sequences (81.98%) exceeded that of duplicated sequences (18.02%), with the lengths of CNVs predominantly ranging between 20 and 500 Kb This distribution demonstrated a decrease in CNVR count with increasing fragment length. Furthermore, an analysis of the population genetic structure using CNVR databases from Chinese, Indian, and European commercial cattle breeds revealed differences between Chinese Bos indicus and Indian Bos indicus. Significant differences were also observed between Hainan yellow cattle and European commercial breeds. We conducted gene annotation for both Hainan yellow cattle and European commercial cattle, as well as for Chinese Bos indicus and Indian Bos indicus, identifying 206 genes that are expressed in both Chinese and Indian Bos indicus. These findings may provide valuable references for future research on Bos indicus. Additionally, selection signatures analysis based on Hainan yellow cattle and three European commercial cattle breeds identified putative pathways related to heat tolerance, disease resistance, fat metabolism, environmental adaptation, candidate genes associated with reproduction and the development of sperm and oocytes (*CABS1*, *DLD*, *FSHR*, *HSD17B2*, *KDM2A*), environmental adaptation (*CNGB3*, *FAM161A*, *DIAPH3*, *EYA4*, *AAK1*, *ERBB4*, *ERC2*), oxidative stress anti–inflammatory response (*COMMD1*, *OXR1)*, disease resistance (*CNTN5*, *HRH4*, *NAALADL2*), and meat quality (*EHHADH*, *RHOD*, *GFPT1*, *SULT1B1*). This study provides a comprehensive exploration of CNVs at the molecular level in Chinese Hainan yellow cattle, offering theoretical support for future breeding and selection programs aimed at enhancing qualities of this breed.

## 1. Introduction

The domestication of livestock has significantly impacted the social and economic dynamics of large populations around the world [1]. This process began with initial domestication of wild ancestral species under natural conditions [2] and progressed through conventional breeding practices involving performance testing and genetic evaluation. These methods have greatly enhanced the productivity of various species. With advancements in genomic sequencing, researchers are now increasingly utilizing animal genomes for breeding purposes. By integrating “biological technologies (BT) + information technologies (IT)”, which combines genomics, computing, and artificial intelligence to analyze biological big data, breeding cycles have been significantly shortened. Recent studies have primarily focused on variations of single nucleotide polymorphisms (SNPs), exploring their associations with economically important traits in various animals such as cattle, sheep, and pigs [2,3,4]. In contrast, another form of genomic variation known as copy number variation (CNV) has not yet gained widespread attention.

CNV, a common form of genomic variation, is characterized by alterations in DNA content and structure that lead to chromosomal rearrangements such as duplications, deletions, inversions, and translocations [5,6]. CNVs typically range in size from 50 base pairs to 5 megabases [7], representing a significant type of genetic variation. The study revealed that 25% of CNVs were not in linkage disequilibrium with the detected SNPs, indicating that CNVs play a distinct and irreplaceable role compared to SNPs [8]. CNVs have been implicated in various evolutionary processes and in the adaptation of organisms to their environments by influencing the emergence of distinct phenotypic traits in both natural and domesticated species. CNVs may explain more individual differences than SNPs in terms of the total nucleotide count involved [9], making them particularly useful for identifying candidate genes related to germplasm traits. For example, researchers have identified several CNV genes associated with lipid transport and fat metabolism in local cattle via whole–genome sequencing [10]. Additionally, other studies have identified CNV segments associated with beef quality traits by analyzing the genome of Chinese Simmental beef cattle [11].

Domestic cattle are categorized into two groups based on their physical characteristics and origins. The first group, Bos taurus have a wild ancestor known as the aurochs (*Bos primigenius*). Aurochs were widely distributed across Europe, Asia, and North Africa. *Bos taurus* cattle are often recognized for their robust build and were traditionally used for plowing and transportation. In contrast, the second group, *Bos indicus*, is characterized by cattle with a prominent hump on their shoulders, primarily originating from South Asia. These humped cattle are especially adept at thriving in hot and humid climates, making them well–suited to regions with challenging environmental conditions [12]. Hainan yellow cattle exhibit prominent shoulder humps and sturdy limbs, as shown in Figure S1. This breed belongs to a subtype of hump cattle in southern China, and it was officially listed in the “Catalog of Chinese Livestock and Poultry Breed Resources Protection” in 2003. Hainan yellow cattle are geographically situated in subtropical regions, and they thrive in environments characterized by high temperatures and humidity, which has led to their notable heat tolerance through natural selection and local environmental adaptation. In addition, Hainan yellow cattle are recognized for traits such as coarse feeding resistance, strong draft power, adaptability, and disease resistance [13]. They are valued for their tender and protein–rich meat, excellent marbling, and high nutritional content, making them highly esteemed by local residents [14]. In recent years, genomic analyses of Hainan yellow cattle have progressively advanced. For instance, Wang et al. (2024) explored the genetic mechanisms behind heat tolerance based on the SNP variation database of Hainan yellow cattle [15]. Chen et al. (2023), through whole–genome SNP analysis, identified genes potentially associated with resilience to environmental pressures such as climate and disease [16]. Liu et al. (2022) conducted ROH analysis based on SNP data [17]. However, reports specifically addressing CNV related to Hainan yellow cattle are scarce. Therefore, exploring copy number variation regions (CNVRs) could reveal genes and gene sets relevant to Hainan yellow cattle. More extensive research is needed on CNV–based exploration of germplasm characteristics and functional enrichment analysis specific to Hainan yellow cattle.

Current research is sparse on CNV–based exploration of germplasm characteristics and functional enrichment analysis specific to Hainan yellow cattle. Hence, CNV has considerable potential for applications in molecular breeding of animals. This study aims to utilize whole–genome sequencing data of Hainan yellow cattle to investigate CNV variations, construct CNVR maps, analyze genomic region density variations across chromosomes, annotate CNVRs, and systematically elucidate genomic segments and pathways related to germplasm traits of Hainan yellow cattle. The expected findings aim to provide valuable insights for advancing CNV research in Hainan yellow cattle at the genomic level.

## 2. Results

### 2.1. Variation Detection and CNV Analysis

The average sequencing depth in this study was approximately 9.17×, resulting in a total of 814.50 Gb of raw data. After our initial quality control filtering, we obtained 812.21 Gb of clean data, with an average Q20 content of 98.83%, average Q30 content of 96.35%, and GC content of 41.81%. Detailed individual sample information is provided in Supplementary Table S1. Subsequent sorting and deduplication procedures identified 197,434 high–quality CNVs and 5852 CNVR segments for Hainan yellow cattle.

**Figure 1:**
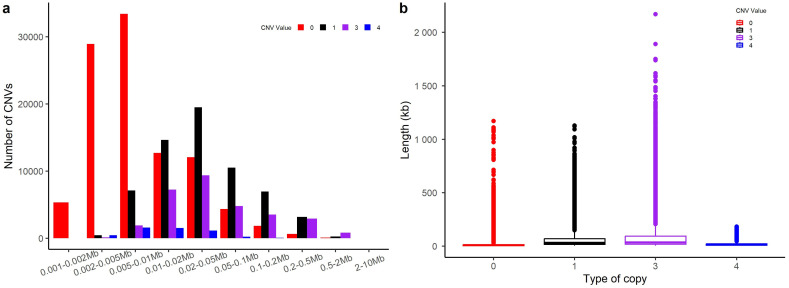
CNV variants across chromosomes. This figure presents a statistical analysis of CNV variant types across chromosomes. In the figure, ‘0’ denotes homozygous deletions (loss of 2 copies), ‘1’ represents heterozygous deletions (loss of 1 copy), ‘3’ indicates copy number gain, and ‘4’ denotes amplification (≥2 copies gain). (**a**) Summary of the total counts of variants for each type by length. (**b**) Boxplot depicting the statistical analysis of the average lengths of variants for each type.

This study conducted a statistical analysis of the types and lengths of CNVs across chromosomes, as shown in Figure 1. Specific details of each statistical measure can be found in Supplementary Table S2. We observed that CNV lengths generally ranged from 20 to 500 Kb, which accounted for 67.01% of detected CNVs and had an average length of 41.77 Kb. The number of CNVs decreased as the lengths exceeded 500 Kb. Additionally, the study performed a statistical analysis on four types of CNV variations, with detailed statistics presented in Supplementary Table S3. These findings revealed that homozygous deletions were the most frequent, with an average length of 16.016 Kb, while copy number gains exhibited the longest average lengths. The shortest segment measured 1.2 Kb and the longest 2169.4 Kb. Overall, deletions outnumbered duplications. Specific counts of CNVs per chromosome are illustrated in Supplementary Figure S2, and the distribution of CNV types across chromosomes is shown in Supplementary Figure S3.

**Figure 2:**
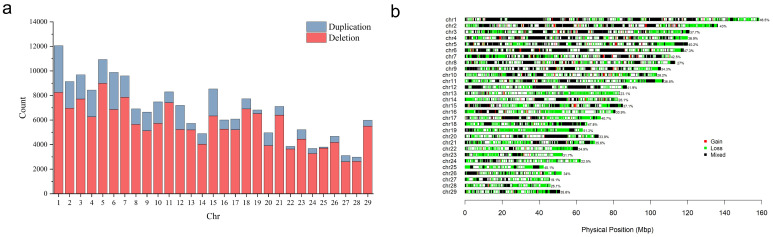
CNV fragment statistics and chromosome distribution of CNVR in Hainan yellow cattle. (**a**) Quantitative analysis of CNV data in Hainan yellow cattle. Red regions indicate deletions, while blue regions represent duplications. (**b**) The distribution of CNVRs across chromosomes in Hainan yellow cattle. ‘Gain’ denotes regions with duplications, ‘Loss’ signifies regions with deletions, and ‘Mixed’ indicates areas where both duplications and deletions are present simultaneously.

Individual CNV segments across each chromosome were systematically analyzed as illustrated in Figure 2a, with detailed statistics provided in Supplementary Table S4. This analysis revealed a higher proportion of deleted sequences (81.98%) as compared to duplicated sequences (18.02%). Chromosome 1 exhibited the highest count of both duplicated and deleted segments. Duplicated sequences contribute additional gene copies, enabling genes to adapt flexibly under selective pressures by losing redundant segments. Over time, mutations and selection can lead to functional changes, either introducing new functionalities or specializing existing ones.

In addition, we also depicted the distribution of CNVRs in the Hainan yellow cattle chromosomes, as shown in Figure 2b, with detailed statistics provided in Supplementary Table S5. This figure illustrates that chromosomes 19, 6, and 1 exhibit higher proportions of CNVRs, potentially due to a greater number of markers located on these chromosomes. Overall, the CNVR markers cover approximately 36.35% of the chromosome lengths collectively.

**Figure 3:**
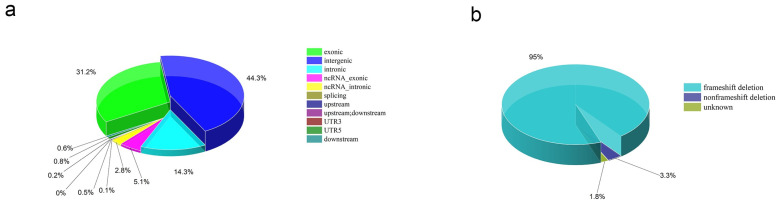
Genomic CNVR annotation information from the Hainan yellow cattle database. (**a**) Genomic CNVR annotation information based on the reference genome, where different colors indicating distinct regions. (**b**) Annotation information of coding regions in Hainan yellow cattle, including frameshift deletions, non–frameshift deletions that do not alter protein coding sequences, and regions of unknown functionality.

To further investigate the genomic characteristics of Hainan yellow cattle, we performed an ANNOVAR analysis to annotate the CNVR segments obtained from the Hainan yellow cattle database. The results, displayed in Figure 3, indicated that CNVRs are unevenly distributed across the genome. Specifically, 44.3% of CNVRs were located in intergenic regions while 31.2% were located in exonic regions. Further analysis of the exonic regions revealed that frameshift deletions accounted for 94.9% of mutations, synonymous mutations (which do not cause changes in protein coding sequences) accounted for 3.3%, and mutations with unknown functions constituted 1.8%. For detailed information, please refer to Supplementary Table S6.

### 2.2. Population Genetic Structure Analysis of Chinese, Indian, and European Commercial Cattle Breeds

**Figure 4:**
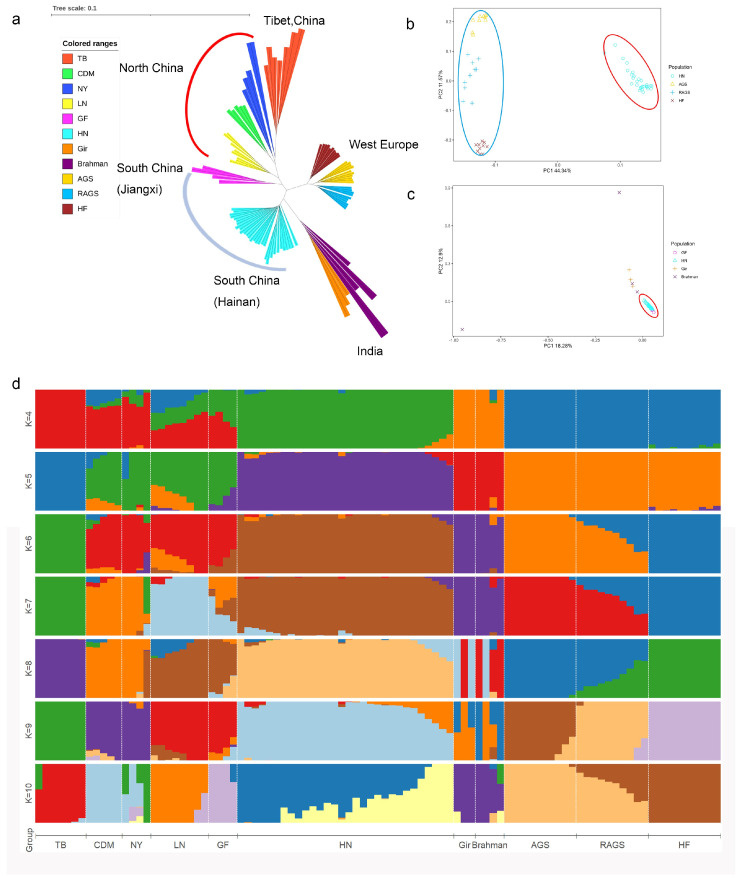
Population genetic structure analysis of Chinese, Indian, and European commercial cattle breeds based on CNVR variations. TB: Tibetan cattle (Tibet, China); CDM: Chaidamu cattle (Qinghai, China); LN: Lingnan cattle (Shanxi, China); NY: Nanyang cattle (Henan, China); GF: Guangfeng cattle (Qinghai, China); HN: Hainan yellow cattle (Qinghai, China); Gir: Gir cattle (Jiangxi, China); Brahman: Brahman cattle (Gujarat, India); AGS: Angus cattle (Aberdeenshire, Scotland, United Kingdom); RAGS: Red Angus cattle (Aberdeenshire, Scotland, United Kingdom); HF: Hereford cattle (Hereford, United Kingdom). (**a**) Neighbor–joining (NJ) tree constructed based on CNVR variations for Chinese, Indian, and European commercial cattle breeds. (**b**) Principal component analysis (PCA) plot showing the distribution of Hainan yellow cattle and European commercial cattle breeds along PC1 and PC2 dimensions. The red circle indicates the clustering of Hainan Yellow Cattle, while the blue circle highlights the clustering of European commercial cattle breeds. (**c**) PCA plot showing the distribution of Chinese and Indian Bos indicus along PC1 and PC2 dimensions. The red circle indicates the clustering of Southern Chinese Bos indicus. (**d**) Population structure inferred using Bayesian inference method (K = 4–10). Different colors represent distinct subgroups.

Based on CNVR variation segments, we analyzed the population genetic structure of Hainan yellow cattle, Chaidamu cattle, Lingnan cattle, Nanyang cattle, Guangfeng cattle, Tibetan cattle, Brahman cattle, Gir cattle, Angus cattle, Red Angus cattle, and Hereford cattle, with results depicted in Figure 4.

The results from the neighbor–joining (NJ) tree analysis indicate that Chinese native cattle cluster separately from European and Indian cattle breeds. Notably, we observed substantial differences among Chinese cattle from the southern, northern, and Tibetan regions, highlighting the complex lineage of East Asian cattle. Southern Chinese cattle are closer to Indian Bos indicus cattle than to their northern Chinese counterparts, and there is a significant separation between Hainan yellow cattle and European commercial breeds. Furthermore, we conducted a principal component analysis (PCA) comparing Hainan yellow cattle with European commercial cattle, as shown in Figure 4b. The PCA, focusing on dimensions PC1 (44.34%) and PC2 (11.57%), revealed a clear separation between Hainan yellow cattle and European commercial breeds, categorizing them into two distinct clusters. To further investigate the differences among Bos indicus, we performed PCA on Chinese and Indian Bos indicus, illustrated in Figure 4c. When analyzing PC1 (18.28%) and PC2 (12.9%), the results indicated that southern Chinese Bos indicus cluster closely together, while Indian Bos indicus form a distinct group, further corroborating the NJ tree analysis. The study also included a structure analysis, which demonstrated that at K = 4, the population can be divided into four groups: one consisting of northern and Tibetan Chinese cattle, another representing southern Chinese cattle, a third group for Indian cattle, and a fourth for European commercial cattle. When K = 5, the Tibetan cattle were isolated, suggesting that the pure East Asian taurine cattle may have been maintained in the Qinghai–Tibet Plateau due to geographical barriers.

In summary, the population structure reveals the adaptive migration history of Hainan yellow cattle, indicating a degree of separation from Indian Bos indicus, particularly increasing towards northern China. This provides a promising avenue for identifying adaptive genes related to Hainan yellow cattle.

Utilizing ANNOVAR for gene annotation based on a reference genome, we identified a total of 1662 genes in the exonic regions of Hainan yellow cattle. Additionally, the exonic regions of Angus cattle revealed 392 annotated genes. Red Angus cattle identified 436 genes, while Hereford cattle identified 625 genes.

**Figure 5:**
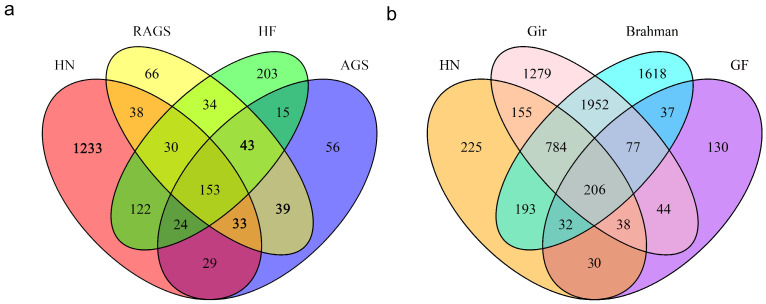
Venn diagram of exonic genes across different cattle populations. (**a**) Hainan yellow cattle and European commercial cattle breeds. (**b**) Bos indicus breeds from China and India. GF: Guangfeng cattle; HN: Hainan yellow cattle; Gir: Gir cattle; Brahman: Brahman cattle; AGS: Angus cattle; RAGS: Red Angus cattle, HF: Hereford cattle.

A Venn diagram (Figure 5a) was used for interspecies comparison, revealing that 1233 genes are present in Hainan yellow cattle but not annotated in European commercial cattle. Detailed gene information can be found in Supplementary Table S7. These findings indicate significant differences in CNVR–encoded genes between Hainan yellow cattle and European commercial cattle breeds, consistent with the results of population structure analysis, thus suggesting further avenues for exploration.

Additionally, we conducted gene annotation for various Bos indicus breeds from China and India, constructing a Venn diagram that identified 206 shared genes (Figure 5b). Within these annotated genes, there may be several candidate genes associated with the formation and adaptability of Bos indicus. Consequently, this study will compare these candidate genes against those identified through selective signal analysis to determine if there are any relevant genetic associations.

### 2.3. Fixation Index (F_ST_) Selection Signatures Analysis

**Figure 6:**
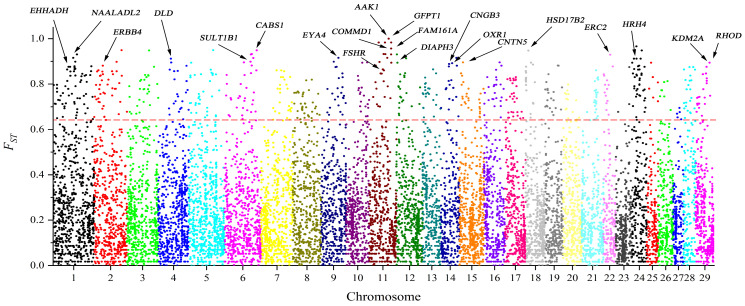
Manhattan plot of Fixation index (*F_ST_*) for positively selected regions within CNVRs of Hainan yellow cattle, with regions above the red line indicating the top 1% under selection. The different colors represent different chromosomes. The red line represents the critical value.

This study conducted a selection signature analysis on CNVRs between the populations of Hainan yellow cattle and European commercial cattle breeds (Figure 6). The top 1% signatures of *F_ST_* were selected for analysis, employing weighted *F_ST_* as a criterion with a critical value set at 0.649. Using the Ensembl database, a total of 96 candidate genes were identified. The detailed names of these candidate genes can be found in Supplementary Table S8. Based on these candidate genes, gene ontology (GO) and Kyoto Encyclopedia of Genes and Genomes (KEGG) pathway enrichment analyses were conducted.

### 2.4. Functional Enrichment Analysis of Candidate Genes

We conducted functional enrichment analysis on 96 candidate genes to investigate their specific pathways, revealing a total of 21 GO terms and 7 KEGG pathways, all listed in Supplementary Table S9. Among these, seven GO terms and three KEGG pathways were found to be significantly enriched (*p* < 0.05). However, the KEGG pathways were not significant after FDR or Bonferroni correction, indicating that there may be false positives. The following three KEGG pathways are enriched (Table 1).

**Figure 7:**
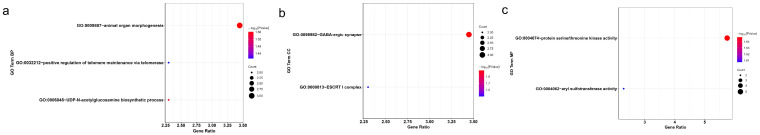
Bubble charts depicting enriched gene ontology (GO) terms based on *F_ST_* –filtered candidate genes. (**a**) Bubble plot of significant GO biological process enrichment analysis based on genes enriched in CNVRs (*p* < 0.05). (**b**) Bubble plot of significant GO cellular component enrichment analysis results based on genes enriched in CNVRs (*p* < 0.05). (**c**) Bubble plot of significant GO) molecular function enrichment analysis results based on genes enriched in CNVRs (*p* < 0.05).

Additionally, among the significant GO terms, biological processes (BP) account for 42.86%, cellular components (CC) account for 28.57%, and molecular functions (MF) account for 28.57%. Figure 7 presents a bubble chart illustrating all GO terms.

## 3. Discussion

Selective pressures commonly imprint distinctive features on the genome, enabling the identification of markers through methods such as selection signatures [18]. Due to the geographical and climatic conditions in Hainan Province, China, Hainan yellow cattle, a local breed, exhibits favorable traits including tolerance to coarse feed, heat resistance, strong draft capability, adaptability, and strong disease resistance, shaped by natural and artificial selection pressures. In recent years, studies on important ruminant species, including cattle, goats, and sheep, have constructed CNV databases, revealing the significant role of CNV variations in the adaptive evolution mechanisms related to environmental changes [19,20]. These findings have important implications for breeding practices. The results of population genomics based on CNVs may provide new insights into the functional and evolutionary research of Hainan yellow cattle [21]. Given the breed’s excellent environmental adaptability, disease resistance, and heat tolerance, as well as its high–quality meat, exploring its CNV data is crucial for breeding efforts. Through previous studies, we recognize that coverage is critical for the reliable detection of CNVs, as higher coverage correlates with increased accuracy in identifying these genomic alterations. Enhanced coverage improves the sensitivity of CNV detection, allowing for the identification of both common and rare CNVs that may play significant roles in adaptive traits. For instance, the results from 10× depth data show similarities to those from 30× CNV identification [10,22]. Additionally, greater sequencing depth reduces the impact of random noise and technical artifacts, thereby decreasing the likelihood of false positives, which is essential for preserving the integrity of CNV data. Therefore, this study, based on 10× depth sequencing, aims to mine genomic information from CNVR databases related to Hainan yellow cattle, investigating genes and associated gene sets linked to their adaptability. This approach seeks to uncover previously unexplored genomic insights that are vital for understanding the adaptation mechanisms of Hainan yellow cattle.

### 3.1. CNV Discovery

In this study, we employed second–generation re–sequencing technology, which provided higher confidence and better resolution in calling CNVs as compared to gene chips [23,24]. As a result, many of the newly identified CNVRs are novel and significantly contribute to the existing knowledge of CNVs in Hainan yellow cattle. CNVs cover larger genomic regions than SNPs and can influence gene function through various mechanisms, including altering gene structure, modifying gene regulation, and exposing recessive alleles [25]. We identified a total of 5852 CNVRs across 30 Hainan yellow cattle, a similar number to other domestic breeds [26]. Additionally, we observed that deletions of CNVs outnumbered duplications, consistent with findings from other related studies [27]. The occurrence of CNVR events involving repetitive sequences may represent an essential step in the formation of cattle breeds, potentially introducing new genomic functionalities that aid species evolution and environmental adaptation [28]. This study further elucidates CNVRs in Hainan yellow cattle, providing a solid foundation for investigating genomic characteristics of this breed.

### 3.2. Population Genetic Structure of CNVRs

This study conducted a population genetic structure analysis of Hainan yellow cattle based on CNVR data. The findings reveal that Guangfeng cattle and Hainan yellow cattle, as native Chinese Bos indicus breeds, exhibit a degree of separation from Indian Bos indicus. While there are some differences between them, the proximity of Chinese Bos indicus to Indian Bos indicus suggests that introgression may have facilitated the rapid adaptation of Indian cattle to humid regions [29]. Research indicates that there have been historical gene introgressions between bovine species found in East Asia and Southeast Asia [30]. In northern China, there are indications of some hybridization trends, while Tibetan taurine cattle appear to have preserved a lineage of pure East Asian taurine cattle. This preservation may be attributed to geographical factors. These observations align with results obtained from whole–genome SNP markers [12], reinforcing the notion that CNVs play a significant role in reflecting evolutionary history. The research provides valuable insights into the population genomics of East Asian cattle, shedding light on their origins and admixture events.

By further exploring the genomic data, it may be possible to identify candidate genes related to adaptability, which could enhance our understanding of how these breeds have evolved in response to their environments. Such insights could have important implications for breeding programs aimed at improving traits that are critical for resilience and productivity in varying climatic conditions.

### 3.3. Breed–Specific Genes in Chinese Hainan Yellow Cattle and GO and KEGG Enrichment Analysis

In this study, we annotated CNVRs in Hainan cattle and European commercial breeds, identifying 1233 genes in Hainan cattle that were not annotated in European breeds, highlighting a significant difference. We also found 206 shared genes between Chinese Bos indicus and Indian Bos indicus, potentially related to their phenotypes and adaptability.

We also conducted selective pressure analysis on CNVRs from both Hainan yellow cattle and European commercial cattle breeds that identified 96 candidate genes from the top 1% of *F_ST_*–weighted data as selection regions. GO and KEGG enrichment analyses based on these genes revealed pathways potentially associated with the breed traits of Hainan yellow cattle. Although seven GO terms and three KEGG pathways were found to be significantly enriched with *p* < 0.05, the results may indicate potential false positives after FDR or Bonferroni correction. Therefore, these pathways can provide valuable insights, and further experiments are needed to determine their actual impact.

In the enrichment analysis of candidate genes in Hainan yellow cattle, we identified bta00140 (steroid hormone biosynthesis). Research indicates that steroid hormones play a role in maintaining focal adhesions in bovine oviduct epithelial cells [31] and significantly influence sexual development [32], suggesting this pathway could be one of the reasons for the high reproductive capacity of Hainan yellow cattle Additionally, we identified enrichment in protein serine/threonine kinase activity (GO:0004674). Studies have shown that the *SGK1* (serum/glucocorticoid–regulated kinase 1) gene encodes a serine/threonine protein kinase. *SGK1* promotes lipid accumulation in cattle by regulating the transcriptional activity of FOXO1 [33], potentially contributing to the superior meat quality of Hainan yellow cattle. GABA–ergic Synapse (GO:0098982) was another GO term identified. Dysfunction in GABAergic circuits is closely linked to neurodevelopmental disorders [34], and these synapses play roles in synapse development, memory formation, and animal behavior [35,36]. This may be related to Hainan yellow cattle heightened nervous sensitivity and adaptability in the wild.

### 3.4. Selection Signature Analysis of Hainan Yellow Cattle Based on Autosomal CNVRs and Identification of Candidate Genes

Compared to Hainan yellow cattle, Angus and Red Angus cattle are less cold–resistant, occasionally exhibit neurotic behavior, are more challenging to manage, and have lower disease resistance. Furthermore, Hainan yellow cattle produce superior meat quality in terms of various volatile flavor compounds and other indicators when compared to Angus beef [37]. In contrast, Hereford cattle demonstrate poor adaptability, with low tolerance to high temperatures, and they are more prone to developing eye cancer during rearing. Additionally, they tend to have shorter lifespans. Therefore, exploring the genomic potential of Hainan yellow cattle could provide valuable insights into their resilience and superior meat quality, offering opportunities for enhancing livestock breeding programs. The *F_ST_* selection signatures found in this study identified genes associated with the germplasm characteristics of Hainan yellow cattle.

Hainan is located in southern China, so cattle experience a hot and humid climate, making robust reproductive capabilities essential for Hainan yellow cattle. In our research, we identified candidate genes associated with reproduction and the development of sperm and oocytes in these cattle. For example, the *CABS1* gene, encoding a calcium-binding protein, may regulate calcium ion concentrations, thereby affecting sperm motility and the acrosome reaction [38], which includes enhanced flagellar activity and sperm maturation [39,40]. These changes are crucial for sperm function. The *DLD* gene encodes an enzyme that plays a key role in cellular energy metabolism and antioxidant responses, both of which are closely related to sperm health and vitality. *DLD* can act as a redox regulator responsible for reactive oxygen species (ROS) production during sperm capacitation [41,42]. *FSHR* plays a critical role in follicular development and maturation; its binding to FSH promotes follicular growth, making it a key gene in folliculogenesis [43,44]. *HSD17B2* is primarily responsible for converting androgen and estrogen precursors into their active forms, reducing ketone hormones to corresponding alcohols, which affects hormone activity and function [45]. Furthermore, the expression of *HSD17B2* is associated with ovarian and testicular function, influencing follicle development and sperm production, thus impacting fertility [46]. *KDM2A*, a regulator of histone modifications, is involved in gametogenesis and embryonic development, with a relationship to sperm production and fertility. Conditional knockout of *KDM2A* leads to complete male infertility, as spermatogenesis ultimately halts during the fertilization stage of meiosis [47,48].

In addition, Hainan yellow cattle exhibit strong adaptability in the wild. Vision and hearing are particularly crucial for survival, as effective sensory perception enhances the animals’ overall productivity. The *CNGB3* gene encodes the β subunit of cyclic nucleotide–gated channels in cone photoreceptors, playing a vital role in phototransduction regulation [49]. This protein is essential for converting light signals into electrical signals, enabling color vision and visual acuity [50]. Mutations in *CNGB3* can lead to various forms of retinal dystrophy, adversely affecting the function and survival of cone photoreceptors [51]. *FAM161A* is another important gene associated with vision, primarily influencing the structure and function of the retina. Mutations in *FAM161A* are linked to certain hereditary retinal diseases, which typically result in progressive vision loss, significantly diminishing survival abilities in the wild [52,53]. The normal expression of *FAM161A* is critical for maintaining the structural integrity and functionality of retinal cells. *DIAPH3* plays a significant role in regulating the assembly and/or maintenance of actin filaments in inner hair cell stereocilia, impacting the morphology and function of hair cells [54]. Abnormal expression of *DIAPH3* can lead to autosomal dominant auditory neuropathy, resulting in hearing loss [55,56,57]. The *EYA4* gene is also crucial for hearing and cochlear function, with research indicating its important connection to hereditary non–syndromic hearing loss [58,59,60], essential for normal cochlear development and function [61]. Furthermore, Hainan yellow cattle demonstrate good resistance to ultraviolet (UV) radiation, and several genes related to UV resistance have been identified. The *AAK1* gene is pivotal in the cellular response to UV radiation and has been found to be associated with hair follicle and hair growth, potentially playing a protective role against UV exposure [62]. *ERBB4* is associated with phenotypic traits related to cattle domestication, influencing pigmentation and participating in melanin synthesis, thus enhancing UV resistance [63]. The *ERC2* gene has been implicated in responses to severe weather conditions involving intense UV exposure [64]. Therefore, the normal expression of these genes likely underpins the exceptional adaptability of Hainan yellow cattle in their natural habitats.

Hainan yellow cattle are recognized for their heat tolerance, an increasingly important trait in breeding due to frequent extreme weather events in recent years. This study has identified genes associated with oxidative stress and anti–inflammatory responses. The *COMMD1* gene plays a crucial role in the regulation of the NF–κB signaling pathway, which is vital for immune responses and inflammation regulation. Research has shown that reduced levels of COMMD protein contribute to prolonged activation of NF–κB [65]. By modulating NF–κB activity, *COMMD1* can influence the expression of various pro–inflammatory cytokines, thereby helping to control inflammation levels within the body [66]. Additionally, the *OXR1* gene encodes a protein primarily responsible for protecting cells from oxidative damage. Heat stress can lead to the generation of excessive ROS, and *OXR1* helps mitigate oxidative stress by regulating the expression of antioxidant enzymes. This function is critical for protecting mammals from oxidative damage [67,68].

Hainan yellow cattle demonstrate strong disease resistance, indicative of a robust immune system that plays a crucial role in maintaining animal health and combating pathogens. This study has identified candidate genes associated with immunity. *CNTN5* plays a significant role in the immune response to infections. It participates in the interactions between neurons and between neurons and other cells, potentially influencing disease susceptibility and contributing to immune regulation [69,70]. *HRH4*, a type of histamine receptor, is primarily expressed in leukocytes and is involved in the activation of dendritic cells and the differentiation of T cells, demonstrating its immune regulatory function [71,72]. *NAALADL2* is associated with immune homeostasis and may participate in regulating immune responses, including cytokine production and cell proliferation [73]. In studies related to cattle, *NAALADL2* has also been found to be involved in immune processes [74].

Moreover, in harsher environments, the meat quality of most animals tends to decline. However, Hainan yellow cattle maintain their excellent meat quality even under challenging conditions. The meat is prized for its tenderness and savory flavor, making it highly esteemed by local residents. The relationship between the *EHHADH* gene and meat quality is primarily reflected in its impact on fat metabolism and muscle development. *EHHADH* influences intracellular triglyceride and fatty acid levels, regulating genes involved in lipogenesis and fatty acid metabolic pathways, thereby improving meat quality [75,76]. This has also been observed in studies involving other cattle [77]. Tenderness is one of the most important meat quality traits. *RHOD* plays a role in the organization of actin filaments and is associated with protein degradation in pigs, which subsequently affects meat quality traits [78]. *GFPT1* is involved in glucose metabolism and is differentially expressed in adipose tissue, playing a significant regulatory role in fatty acid metabolism and fat deposition [79,80]. Lastly, the relationship between the *SULT1B1* gene and meat quality fat is mainly reflected in fat metabolism and the synthesis of flavor compounds [81].

By delving into the CNV information of Hainan yellow cattle, researchers can gain a deeper understanding of their genetic makeup and enrich the breed’s genomic map. Furthermore, the population structure analysis based on CNV variation data aligns closely with SNP data results, suggesting that it may serve as a vital basis for understanding domestication and evolutionary processes. The identification of candidate genes related to heat tolerance, adaptability, and fertility is particularly valuable for future breeding programs. This knowledge can lead to the development of cattle that are more resilient to environmental challenges, more adaptable to varying conditions, and potentially more productive in terms of reproductive performance. Such advancements can significantly enhance the efficiency and sustainability of the cattle industry, improving livestock management practices and contributing to the overall economic viability of the sector. However, this study has certain limitations, as it did not integrate specific phenotypic data. Future research could benefit from a multi–omics approach, such as combining transcriptomics and metabolomics, to better validate the findings of this study.

## 4. Materials and Methods

### 4.1. Sample Collection and Sequencing

We collected a total of 30 ear tissue samples from Hainan yellow cattle on farms in Hainan, comprising 15 bulls and 15 cows with no familial relationships. The samples were preserved in polypropylene (PP) tubes containing 75% ethanol with added anti–degradation agents and briefly stored on dry ice before being sent to Nuohe Zhiyuan Biotechnology Co., Ltd. for sequencing within 2 h. DNA was extracted from each sample and subjected to a thorough quality assessment. The DNA was then fragmented into random sizes using a Covaris ultrasonic disruptor. Following fragmentation, the DNA underwent end repair to address any damaged ends, and A–tailing was performed to add a single adenine base to the 3′ ends of the fragments. Sequencing adapters were then ligated to both ends of the DNA fragments. Fragment size selection was conducted to isolate DNA fragments of appropriate lengths. The selected fragments were amplified through PCR to generate sufficient quantities for sequencing and purified to remove contaminants. The resulting DNA libraries underwent rigorous quality checks to confirm the absence of contamination. Finally, the libraries were subjected to sequencing using the Illumina next–generation sequencing platform, providing high–resolution genetic information for the study.

### 4.2. Data Quality Control and CNV Detection

We based our analysis on sequencing data from 30 Hainan yellow cattle and incorporated data from the following breeds for comparison: Chaidamu cattle (5, from Qinghai, China), Lingnan cattle (8, from Shanxi, China), Nanyang cattle (4, from Henan, China), Guangfeng cattle (4, from Jiangxi, China), Tibetan cattle (7, from Tibet, China), Brahman cattle (4, from West Bengal, India), Gir cattle (3, from Gujarat, India), Angus cattle (10, from Aberdeenshire, Scotland, UK), Red Angus cattle (10, from Aberdeenshire, Scotland, UK), and Hereford cattle (10, from Hereford, UK). The specific SRA accession numbers used in this analysis are detailed in Supplementary Table S10. To ensure data quality, we performed preliminary quality control on sequencing data. The preliminary data filtering employed the fastp software (v0.2.1) [82] (https://github.com/OpenGene/fastp, accessed on 5 November 2024), with setting quality thresholds for trimming and a minimum sequence length threshold to filter out fragments shorter than 50 bp, and specifying that sequences with more than 10 ambiguous bases (N bases) would be filtered out. The other parameters were set to default values. Subsequently, we used the “MEM” algorithm in BWA (v0.7.17) [83] to align the data to the cattle reference genome (ARS–UCD2.0, https://www.ncbi.nlm.nih.gov/datasets/genome/GCF_002263795.3/, accessed on 5 November 2024) for further analysis.

The SAMtools software (v1.6) [84] sort tool was utilized to convert Sequence Alignment/MAP format (sam) files to Binary Alignment Map (BAM) files and perform sorting, followed by deduplication using sambamba (v0.8.2) [85] and Picard (v3.0) software [86]. Following the same approach by Gao et al. (2017) [87], the obtained Variant Call Format (VCF) files underwent initial filtering, and CNVnator [88] was run on merged BAM files with a bin size of 200 bp to ensure data accuracy. The parameters were set as follows: –his 200–stat 200–partition 200–call 200. Further filtering using Bcftools software (v1.13) [89] included criteria settings including *p*-value < 0.001, length > 1 Kb, and q0 (mapping quality zero) < 0.5, and the CNVs in unplaced scaffolds were removed.

CNV data for each individual yellow cattle were collected, and CNV regions (CNVRs) were identified by overlapping individual CNVs within each breed, considering overlaps of at least 1 bp. The analysis was then confined to CNVRs that were present in at least four individuals (13.3%) [90]. We used the Bcftools software to merge the final CNVR databases for both Hainan yellow cattle and European commercial cattle breeds for further analysis. Beagle software (v5.4) [91] includes modules for related individual phasing and LD phasing, which phase and impute the data. In this study, Beagle was used with the following parameters: imp–segment = 6.0, window = 40.0, and overlap = 4.0. The imputed data were then used to construct CNVR databases for Hainan yellow cattle, Chaidamu cattle, Lingnan cattle, Nanyang cattle, Guangfeng cattle, Tibetan cattle, Brahman cattle, Gir cattle, Angus cattle, Red Angus cattle, and Hereford cattle.

### 4.3. Detection and Statistical Analysis of CNVs in Hainan Yellow Cattle

The number of duplications and deletions within CNV segments across each chromosome was tabulated from the VCF files of Hainan yellow cattle. These counts were then graphically represented to illustrate CNVR duplication and deletion frequencies. In order to visualize CNV distribution across chromosomes, the VCF files were converted into a format recognized by the HandyCNV package (v1.1.7). Using the HandyCNV package’s ‘cnv_visual’ tool, we visually depicted the CNVR distributions across chromosomes. Additionally, the ‘cnv_summary_plot’ parameter was utilized to summarize the CNVR results by length group, CNVR type, chromosome, and individual aggregation in Hainan yellow cattle. Additionally, the ANNOVAR software (version: 7 June 2020) [92] was employed to annotate gene information for CNVR segments identified in Hainan yellow cattle and to produce corresponding charts and figures.

### 4.4. Population Genetic Structure Analysis of Cattle Breeds from China, India, and Europe

Using MEGA software (v7.0) [93], we constructed a neighbor–joining (NJ) tree based on the genetic distance matrices derived from Chinese, Indian, and European commercial cattle breeds. The tree was then visually enhanced using the iTOL website (https://itol.embl.de, accessed on 5 November 2024) [94]. In parallel, PCA of Hainan yellow cattle and European commercial cattle was conducted using Plink software (v1.90) [95]. Additionally, PCA analysis was performed for Chinese Bos indicus and Indian Bos indicus. The clustering, based on PC1 and PC2, was visualized using ggplot2 (v3.5.1) from the R package (v4.2.1). Furthermore, genetic data from CNVR markers specific to the molecular genetic databases of Chinese, Indian, and European cattle breeds were utilized to assess the population genetic structure using ADMIXTURE software (v1.3.0) [96]. Assuming the presence of multiple subgroups within each population, the differences between the various populations were investigated, with different colors representing the various subgroup components.

### 4.5. Selection Signatures Analysis of CNVR in Hainan Yellow Cattle and European Commercial Cattle Breeds

Using VCFtools software (v0.1.16) [97], the genetic differentiation index (*F_ST_*) between the two populations based on CNVR was calculated using the ‘–weir–fst–pop’ command. *F_ST_* values range from 0 to 1 and indicate genetic differentiation; higher values signify greater differentiation whereas lower values suggest lesser differentiation between the populations in CNVRs. In this study, we considered genomic regions corresponding to the top 1% *F_ST_* values as regions under selection. Visualization of these regions was carried out with ggplot2 from the R package, and gene annotation was performed using the Ensembl database (https://useast.ensembl.org/index.html, accessed on 5 November 2024).

### 4.6. Functional Enrichment Analysis of Candidate Genes Associated with CNVRs in Hainan Yellow Cattle

The DAVID (v6.8) database (https://david.ncifcrf.gov, accessed on 5 November 2024) [98] is a bioinformatics resource that integrates biological data and analysis tools. Based on information from the DAVID database, functional enrichment analysis was conducted on genes annotated within the top 1% of genomic regions to identify pathways potentially associated with phenotypic traits to Hainan yellow cattle.

## 5. Conclusions

This study explored CNV variations in Hainan yellow cattle based on whole–genome sequencing data. The average length of CNVs was 41.77 Kb, with deletions being more prevalent than duplications. The study classified the CNVs into four categories for statistical analysis. Additionally, the research revealed CNVR information to this breed, identifying a total of 5852 CNVRs, which account for 39.32% of the genome. This enriches the genomic database of Chinese cattle and provides a foundation for future functional CNV studies and their application in breeding. Additionally, a comparison of the CNVR databases among Chinese, Indian, and European commercial cattle revealed three distinct clusters. Notably, southern Chinese cattle are closer to Indian Bos indicus than to northern Chinese cattle. We identified genes associated with traits that differ between the Bos indicus and Bos taurus breeds, and also between traits unique to Hainan yellow cattle and European commercial breeds. Using selection signatures, this study establishes a theoretical foundation for further research and supports targeted breeding efforts for Hainan yellow cattle. However, the study has limitations, including the need for greater integration with multi–omics data and the lack of further validation of the candidate genes. Deeper sequencing depths may uncover more detailed CNVs, suggesting directions for future research.

## Figures and Tables

**Table 1:** Enriched Kyoto Encyclopedia of Genes and Genomes (KEGG) pathways of candidate genes.

Term	Gene.ratio	*p*-Value	Genes
KEGG_PATHWAY	bta00140:Steroid hormone biosynthesis	0.004481625	*SULT1E1*, *UGT1A1*, *HSD17B2*, *UGT2A1*
KEGG_PATHWAY	bta01240:Biosynthesis of cofactors	0.030034677	*UGT1A1*, *UGT2A1*, *COQ7*, *DLD*
KEGG_PATHWAY	bta04390:Hippo signaling pathway	0.032063576	*PPP1CB*, *PATJ*, *FZD2*, *PARD6G*

## Data Availability

The raw data used in this study are publicly available and can be obtained upon reasonable request to the corresponding author.

## Supplementary Materials

The following supporting information can be downloaded at: https://www.mdpi.com/article/10.3390/ijms252211919/s1.
