# Supplementary figures and images for: Insights into Adaption and Growth Evolution: Genome–Wide Copy Number Variation Analysis in Chinese Hainan Yellow Cattle Using Whole–Genome Re–Sequencing Data
Source: Int J Mol Sci. 2024 Nov 6;25(22):11919. doi: 10.3390/ijms252211919 (PMC11594005; doi:10.3390/ijms252211919)

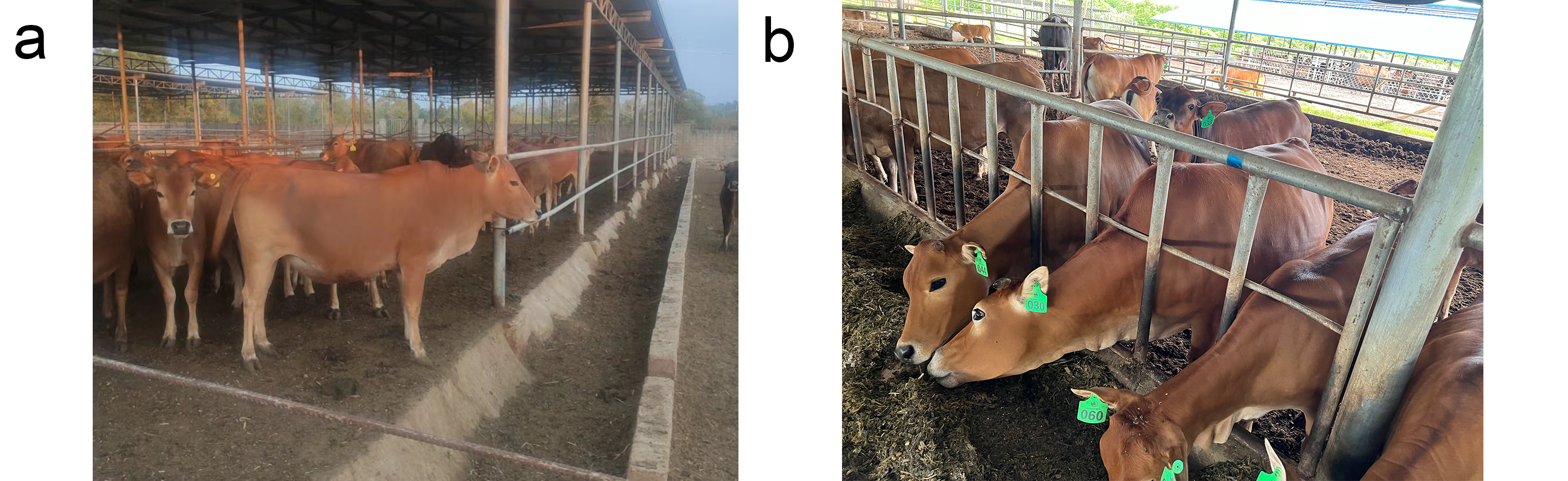

Supplement: Supplementary file 1 [file ijms-25-11919-s001.zip › Figure S1.tif]
